# Patient and Therapist Perceptions of a Publicly Funded Internet-Based Cognitive Behavioral Therapy (iCBT) Program for Ontario Adults During the COVID-19 Pandemic: Qualitative Study

**DOI:** 10.2196/50113

**Published:** 2024-02-19

**Authors:** Serena Thapar, Megan Nguyen, Bilal Noreen Khan, Roz Fanaieyan, Vanessa Kishimoto, Rebecca Liu, Blanca Bolea-Alamañac, Marisa Leon-Carlyle, Anne O’Riordan, Maggie Keresteci, Onil Bhattacharyya

**Affiliations:** 1 Institute for Health System Solutions and Virtual Care Women's College Hospital Toronto, ON Canada; 2 Department of Psychology McGill University Montreal, QC Canada; 3 Institute of Health Policy, Management and Evaluation University of Toronto Toronto, ON Canada; 4 Department of Psychiatry University of Toronto Toronto, ON Canada; 5 Patient Advisors Network Toronto, ON Canada; 6 Department of Family and Community Medicine University of Toronto Toronto, ON Canada

**Keywords:** depression, anxiety, cognitive behavioral therapy, digital health, internet-delivered cognitive behavioral therapy, iCBT, CBT, implementation, facilitators, barriers, interviews, qualitative

## Abstract

**Background:**

To address the anticipated rise in mental health symptoms experienced at the population level during the COVID-19 pandemic, the Ontario government provided 2 therapist-assisted internet-delivered cognitive behavioral therapy (iCBT) programs to adults free of charge at the point of service.

**Objective:**

The study aims to explore the facilitators of and barriers to implementing iCBT at the population level in Ontario, Canada, from the perspective of patients and therapists to better understand how therapist-assisted iCBT programs can be effectively implemented at the population level and inform strategies for enhancing service delivery and integration into the health care system.

**Methods:**

Using a convenience sampling methodology, semistructured interviews were conducted with 10 therapists who delivered iCBT and 20 patients who received iCBT through either of the publicly funded programs to explore their perspectives of the program. Interview data were analyzed using inductive thematic analysis to generate themes.

**Results:**

Six salient themes were identified. Facilitators included the therapist-assisted nature of the program; the ease of registration and the lack of cost; and the feasibility of completing the psychoeducational modules given the online and self-paced nature of the program. Barriers included challenges with the online remote modality for developing the therapeutic alliance; the program’s generalized nature, which limited customization to individual needs; and a lack of formal integration between the iCBT program and the health care system.

**Conclusions:**

Although the program was generally well-received by patients and therapists due to its accessibility and feasibility, the digital format of the program presented both benefits and unique challenges. Strategies for improving the quality of service delivery include opportunities for synchronous communication between therapists and patients, options for increased customization, and the formal integration of iCBT into a broader stepped-care model that centralizes patient referrals between care providers and promotes continuity of care.

## Introduction

The COVID-19 pandemic presented a significant challenge to the mental health of Canadians [1]. Studies have drawn attention to the negative impact of the pandemic on population-level mental health, highlighting increased levels of self-reported symptoms of depression and anxiety among Canadian adults [2-4]. Research conducted throughout the pandemic demonstrated barriers to accessing in-person mental health care in Canada, which occurred in the context of existing challenges, including lengthy wait times, limited availability of services in rural areas, high cost of services, a shortage of mental health professionals, stigma, and a lack of integration of mental health care services [5]. Due to the shortage of trained mental health professionals in Canada, the current workforce is unable to adequately meet the demands of one-on-one in-person psychotherapy [6]. Therefore, other solutions are required.

Digital mental health interventions, such as internet-delivered cognitive behavioral therapy (iCBT), are promising because they may address many of these barriers to care by providing increased reach at a lower cost than other modalities, subsequently improving access to evidence-based mental health treatment for depression and anxiety [7]. iCBT involves structured and predefined psychotherapeutic content organized in online modules. Patients are assigned homework to consolidate their learning, and brief therapist support is typically provided via secure in-app messaging and calls [8]. Randomized controlled trials have provided evidence in support of the effectiveness of iCBT across psychiatric disorders [9,10]. For example, a meta-analysis of transdiagnostic iCBT found medium to large controlled effect sizes for depression and anxiety outcomes (*g*=0.79 to 0.82) [11]. The treatment has also been successfully implemented in routine care settings in countries such as Australia, Sweden, Denmark, and Norway [12]. Despite its apparent efficacy and efforts to reduce barriers, iCBT programs face difficulties in maintaining user engagement, which is evident by limited uptake and high dropout rates [13]. Dropout in digital mental health therapies has been a long-standing issue, with dropout rates being typically lower in study trials compared with real-world implementations and routine care [14]. For example, some studies report that just more than half of patients complete a full course of iCBT [15]. A meta-analysis of 7313 participants across 40 studies found a dropout rate of 57%, with higher rates of dropout for iCBT programs for depression without support (74%) and lower rates for those with therapeutic support (28%) [16]. Real-world iCBT implementations in Australia and the United Kingdom (eg, This Way Up, MindSpot, and Improving Access to Psychological Therapies) show dropout rates ranging from 29% to 64% [17-19]. Future work should focus on identifying participants who will most likely benefit from iCBT and adhere to treatment protocols.

In addition, studies have shown that iCBT is a cost-effective way to improve accessibility to care, particularly for patients who face geographic and mobility limitations, such as those who live in remote or rural areas [20]. Moreover, Health Quality Ontario has suggested that the few publicly funded psychotherapy services in Ontario are not equitably distributed across the province, such as in rural areas in Northern Ontario where there are fewer psychiatrists [21], and cannot meet the needs of patients with mental health concerns [9]. Indeed, there is strong evidence that there is income-based inequity in access to mental health services, especially for psychologists who tend to be concentrated in private practice under the existing 2-tiered mental health care system in Canada [22,23]. Mental health services provided by general practitioners and psychiatrists can be billed through provincial and territorial health insurance plans; however, services offered by allied health professionals, such as psychologists, cannot. Estimates suggest that while two-thirds of Canadians can access mental health services through private employer benefits, the remaining one-third must access services through limited publicly funded services, pay out-of-pocket, or forgo seeking care [24]. Therefore, leveraging digital health technology can increase patient access to evidence-based psychotherapy, regardless of whether or not there is a pandemic.

To address the anticipated rise in mental health symptoms experienced at the population level, the Ontario government rapidly expanded digital mental health service offerings in May 2020 [25]. This included therapist-assisted iCBT, a form of guided iCBT where licensed mental health professionals provide regular support for patients by monitoring their symptoms, offering regular check-ins, and giving feedback on their homework assignments. This form of iCBT can be contrasted to coach-assisted iCBT, where nonregulated mental health workers are trained to provide support to patients throughout the program, or self-guided iCBT, where patients access a series of modules independently.

While publicly funded iCBT has been successfully implemented in Ontario during the pandemic by 2 service providers (ie, privately owned Canadian companies in the mental health and wellness sector), patient and therapist perceptions around its implementation have yet to be explored. Research on the experience of patients and therapists with publicly funded iCBT in other jurisdictions highlights accessibility, convenience, and the role of therapist support [26,27]. While these findings provide valuable insights, it is important to acknowledge potential differences that cultural factors, health care systems, and program variations may influence. Furthermore, few studies have examined the uptake and experience of publicly funded iCBT programs in Canada [28]. Therefore, the study objectives were to explore, from the perspectives of patients who accessed the iCBT program in Ontario and the therapists who provided the service, the facilitators and barriers related to iCBT delivery during the pandemic, and the proposed recommendations to address such barriers. The findings can inform future policy and funding decisions by providing insight into whether and how the service should be expanded and better integrated into the mental health care system.

## Methods

### Study Setting

This study aimed to investigate the therapist-assisted iCBT program funded by the Ontario government during the COVID-19 pandemic to residents and offered in both English and French [25]. The 10- to 12-week program was promoted to individuals experiencing mild to moderate depressive or anxiety symptoms. The program could be accessed through self-referral or clinician referral to 1 of 2 service providers. Both service providers offered programs that adhered to the general principles of iCBT programs, which include a comprehensive intake assessment to identify primary mental health concerns, cognitive behavioral therapy techniques, and ongoing access to communication with a regulated mental health professional [29]. Both programs were based on the same principles but differed in their intake assessment process and method of communication between the therapist and patient. Program A (LifeWorks AbilitiCBT) offered a 5- to 7-minute online intake questionnaire, followed by a mandatory remote synchronous intake assessment with a therapist to identify the patient’s mental health needs and to develop a protocol tailored for the patient’s primary mental health concern [30]. Program B (Mind Beacon TAiCBT) used a 30-minute online intake questionnaire to achieve the same aims [31]. Both programs had a secure in-app messaging system for communication between patients and therapists, through which therapists could send messages to clients derived from a bank of predetermined messages. However, only program A offered optional synchronous phone or video calls that could be scheduled if needed throughout the program. Program A provided most patients with 10 content modules, irrespective of their specific mental health needs. One exception was for patients with posttraumatic stress disorder or trauma, who were provided with 2 additional modules for a total of 12 modules and an estimated completion time of 10 weeks. Program B provided patients with an average of 7 to 16 playlists (synonymous with modules), with additional playlists of content provided depending on the individual- or condition-specific needs, for an overall average completion time of 12 weeks. Despite the slight programmatic differences, it is reasonable to argue that the barriers to and facilitators of uptake and engagement in iCBT are unlikely to differ significantly between the 2 programs, both of which were implemented in the same context and aim to provide accessible and convenient iCBT interventions using online platforms and secure messaging system for patient-therapist communication. By conducting a combined analysis, a larger data set can be obtained, enabling a broader perspective on barriers and facilitators to iCBT implementation at the population level and the identification of overarching themes that go beyond program-specific differences. By focusing on common factors among programs, the study findings can provide valuable insights for the development and improvement of implementing iCBT programs across contexts.

### Study Design

In this qualitative study, the research coordinator (MN) and 2 research assistants (BNK and ST) conducted semistructured, emergent interviews [32] with patients and therapists to gain a thorough understanding of their perspectives and experiences using or delivering iCBT. All interviewers had prior experience conducting semistructured interviews and received training prior to data collection. The study design and interview guides were developed based on input from multiple stakeholders, including patient advisors, psychiatrists, and experts in qualitative research. Furthermore, the interview guides were pilot-tested among the research team. The initial interview questions aimed to elicit contextual information, understand acceptance and satisfaction with the program, identify barriers and facilitators in using or delivering iCBT, and gather feedback on refining the service for future offerings. The interviews also focused on gaining insight into program completion and communication between therapists and patients. Follow-up questions were then tailored to participants’ responses to the initial questions in keeping with the emergent interview design. Finally, to ensure the relevance of the questions, separate interview guides were developed for patients and therapists (see Multimedia Appendix 1).

### Recruitment and Data Collection

The 2 service providers assisted with recruitment by sending emails and in-app messages on behalf of the research team to the therapists and patients enrolled in the program. Both service providers sent recruitment emails to all therapists on an internal listserve and in-app message prompts through the platform to reach all users with access to the program on a weekly basis between September and November 2021. During the recruitment period, 39 patients (20 in program A and 19 in program B) and 29 therapists (17 in program A and 12 in program B) contacted the research team via email expressing interest in the study. Between October and December of 2021, 30 interviews were conducted with 20 patients (10 in program A, 9 in program B, and 1 in both program A and program B) and 10 therapists (5 in program A and 5 in program B). Interviews were conducted until data saturation was reached.

The interviews were conducted between the interviewer and interviewee who had no relationship established prior to study commencement via audio call using Microsoft Teams and lasted approximately 45 minutes. Inclusion criteria for patients included being 18 years or older, having mild to moderate depression- or anxiety-related symptoms at the time of registration with the iCBT program, and having accessed the publicly funded program during the COVID-19 pandemic (between May 2020 and December 2021). The inclusion criteria for therapists included being a health care professional who delivered the iCBT program from either provider during the COVID-19 pandemic.

### Data Analysis

The interviews were audio recorded, transcribed verbatim, and analyzed using inductive thematic content analysis, allowing for the emergence of themes and patterns directly from the data [33]. Transcripts were not returned to participants for comment or correction, nor did participants provide feedback on the findings. MN, RF, ST, and VK all contributed to the data coding and analysis process, which involved iteratively reading and rereading the transcripts to identify meaningful patterns and themes. This data-driven approach ensured that the analysis was grounded in the participants’ experiences and perspectives. To ensure rigorous methodology, the research team engaged in multiple meetings to develop consensus and refine the emergent themes.

To analyze the data, the researchers first independently reviewed the transcripts and created initial codes. Together, they then confirmed the structure of the codes and looked for potential themes. Once themes were identified, they were named and defined, using exemplar quotes to illustrate key points. All research team members reviewed and provided input on the themes and their interpretations. The themes and codes were related back to the study objectives during paper preparation. To ensure rigor and trustworthiness, the researchers engaged in reflexivity and debriefing with peers throughout the analysis [34].

### Ethical Considerations

The study received ethical approval from the Women’s College Hospital Ethics Assessment Process for Quality Improvement Projects (REB # 2021-0057-E). All participants provided written and verbal consent before participating in the semistructured interview, during which the research objectives were described. Participants were made aware that the interviewers were unbiased third-party evaluators of the program. All participants were compensated via a $25 CAD electronic gift card to their choice of one of several major retailers. The data has been de-identified.

## Results

### Demographics

Table 1 displays the demographic characteristics of the patient interviewees. Of the 20 patients, most (n=13, 65%) were between the ages of 20 to 50 years; 55% (n=11) of patients were female and 40% (n=8) of patients were male. Regarding ethnicity, 70% (n=14) of the patients identified as White, while 30% (n=6) of patients identified as belonging to a racialized minority group. Notably, 80% (n=16) of patients completed 10 or more modules or playlists, meaning that they completed most of the program content without dropping out prematurely. A total of 25% (n=5) of patients had an average comfort with technology, while 30% (n=6) and 45% (n=9) of patients reported advanced and expert comfort with technology, respectively. Furthermore, all patients were self-referred to the program. Many patients heard about the program through a self-directed web-based search (n=9, 45%) or via their social network (n=9, 45%), while only 10% (n=2) of patients heard about the program via advertisements.

Table 2 displays the demographic characteristics of the therapist interviewees. Of the 10 therapist interviewees, most were (n=6, 60%) between the ages of 20 to 35 years, with a higher proportion of female therapists (n=9, 90%). Most therapists were licensed social workers (n=8, 80%), while the remaining 20% (n=2) were registered psychotherapists. Half (n=5, 50%) of all therapists had been delivering iCBT for less than 6 months. Most therapists (n=6, 60%) had 5 years or less of experience in their current profession.

Facilitators of iCBT included the therapist-assisted nature of the program, the ease at which the service could be registered for and accessed on an ongoing basis due to a lack of cost, and the feasibility of completing the psychotherapeutic content given the online and self-paced nature of the program. However, the study identified 3 barriers to the program’s implementation: challenges with the online delivery of the program for the therapeutic alliance; the program’s generalized nature, which limited customization to individual needs; and a lack of formal integration between the iCBT program and the health care system.

**Table 1:** Patient interviewee demographics.

Demographic variables and categories		Number of interviewees (n=20), n (%)
**Age group (years)**		
	20-35	4 (20)
	36-50	9 (45)
	≥51	7 (35)
**Sex**		
	Male	8 (40)
	Female	11 (55)
	Prefer not to answer	1 (5)
**Race or ethnicity**		
	White	14 (70)
	Racialized minority	6 (30)
**Education**		
	High school	2 (10)
	College degree or diploma certificate	9 (45)
	Undergraduate degree or above	8 (40)
	Prefer not to answer	1 (5)
**Number of modules or playlists completed**		
	6-10	4 (20)
	>10	16 (80)
**Comfort with technology**		
	Average	5 (25)
	Advanced	6 (30)
	Expert	9 (45)
**How patients heard about the program**		
	Self-directed web-based search	9 (45)
	Social network or care provider	9 (45)
	Advertisement	2 (10)

**Table 2:** Therapist interviewee demographics.

Demographic variables and categories		Number of interviewees (n=10), n (%)
**Age group (years)**		
	20-35	6 (60)
	36-65	4 (40)
**Sex**		
	Male	1 (10)
	Female	9 (90)
**Professional designation**		
	Social worker	8 (80)
	Registered psychotherapist	2 (20)
**Years in current profession**		
	5 years or less	6 (60)
	>6 years	4 (40)
**Length of time delivering iCBT^a^**		
	6 months or less	5 (50)
	More than 6 months	5 (50)

^a^iCBT: internet-delivered cognitive behavioral therapy.

### Facilitators of iCBT

#### The Therapist-Assisted Nature of the Program

Patients valued the therapist-assisted nature of the iCBT program, with many reporting satisfaction with being able to connect with a mental health professional. Personal communication with and feedback from therapists were essential in supporting patients’ engagement with the program and creating accountability for completing the program content, as they regularly checked in on patient progress.

I was really busy and I kind of left it unattended, and then [my therapist] just told me...don’t give up, find the time even though it might be busy, just take time aside and keep going, you’re almost done. I think if she wouldn’t be there or she wouldn’t send me that message, I would probably [have] just dropped it.

Patient 24, program A

I will get occasional emails, “Your therapist has checked in”...it’s almost like a cue that, “Are you on track with your lessons?” Because I think if you leave people to their own devices, sometimes they’ll just fall off...the only accountability is to yourself.

Patient 7, program B

In addition, the presence of a therapist allowed patients to feel connected to another human despite the online nature of the program. Patients felt supported knowing that an individual cared about their progress, read their messages, and reviewed their work.

I think I also don’t want to just feel so clinically cold about it either...that I just do my thing, and hopefully something will come of it. It’s nice for me to know that there’s somebody checking in...you feel like somebody cares. I kind of like the messaging, I think that without it, it’s a little sterile.

Patient 7, program B

I felt like somebody was actually listening to me. I wasn't just going through a computer program.

Patient 24, program A

As several patients noted, the presence of a therapist was perceived as the most valuable component of the program.

I felt the material that I was working on was helpful, but the therapist was most helpful for what was there...I don’t think it would have been of any value to me personally without a therapist involved...For me that was one of the biggest things, was just to have someone to lean on a little bit.

Patient 13, program B

That’s the best part...the conversation with the therapist.

Patient 2, program A

However, as 1 therapist noted, not all patients take advantage of the ability to engage with a therapist.

Several patients appreciated the asynchronous communication modality, which allowed for a more accessible introduction to mental health treatment for those reluctant to engage in traditional therapy formats.

It makes you not quite so vulnerable as one-on-one counselling either on the phone or face-to-face, for somebody who is new to it or may have been reluctant in the past, I think that was the initial appeal for me, was that there was that kind of buffer...having that initial message from your e-therapist...was a very gentle introduction to therapy.

Patient 11, program B

Other patients similarly noted that they felt more comfortable openly expressing personal topics through messaging as opposed to face-to-face or over the phone (patient 7, program B).

#### The Accessibility of Registering for the Program

The iCBT program was found to be accessible for patients due to its free and immediate availability. Many patients interviewed were willing to enroll in the iCBT program because it was available almost immediately with no waiting period.

As soon as I finished my [intake assessment], I got the email almost immediately...within a week, I had a therapist send me an email to set up our initial interview.

Patient 28, program A

The lack of cost enabled individuals experiencing financial difficulties to participate, as well as those who were on waitlists or could not afford mental health services. As 1 therapist noted, the low barrier to entry is especially important because “so many people are looking for support...on waitlists...having to pay out of pocket, [and] are feeling they can’t find anyone to talk to...This is a great base for them to get that step in the door to build that confidence to be able to get more support” (therapist 4, program A). For example, 1 patient noted that they were interested in pursuing traditional CBT but did not have the resources to do so. The program being offered free of charge reduced the barrier to engagement.

Doctors thought that CBT was the appropriate therapy for me, and so...I would go through an evaluation, and then it was a horrendous cost which I couldn’t afford...The cost is prohibitive.

Patient 26, program A

For many patients, money is a factor in their ability to access mental health services. As 1 patient stated, “I would have had to look at my benefits to see if something else was covered, but when I read that [it was free], I knew that it was not going to be a financial burden on me.” (patient 13, program B). However, some therapists expressed concern that the free service made it noncommittal and may contribute to patient withdrawal or inactivity.

#### The Feasibility of Completing the Psychotherapeutic Content of the Program

Furthermore, the online nature of the program also allowed for easy access from home or mobile devices, making it more feasible.

It was the most practical thing ever. Everybody sits on their phone and scrolls through Instagram and Facebook and stuff, so having the module accessible on my phone was amazing because I was able to do that at any point in time throughout my day.

Patient 28, program A

One patient noted that they had to travel outside the home to receive the services, they would not have been able to participate (patient 26, program A). Indeed, the accessibility of the program was appealing to many patients who were not generally available during business hours. For example, 1 patient noted that the online delivery “makes the service much more accessible because people who are busy or working...it makes it more feasible to access the program compared with having to go into a doctor's office” (patient 6, program A). This was especially important for patients who did not feel comfortable leaving their homes during the pandemic or lacked access to transportation. As 1 therapist noted, the program makes it easier for patients to access mental health support because it reduces the effort required to attend in-person therapy, a step which may compound the existing difficulties for patients experiencing challenges with their mental health.

Patients also appreciated the ability to work at their own pace and revisit their worksheets following the completion of the program. Patients similarly were satisfied with always having the program available.

I really enjoyed the immediate accessibility of it...just having the reassurance that it is immediately at hand is oftentimes, sometimes all you need.

Patient 11, program B

Other patients noted the unique ability to spread out their therapy over time and work at their own pace.

I would just get up and walk away or maybe go for a walk and come back and you can’t do that in traditional therapies.

Patient 26, program A

As 1 therapist noted, “I feel like one of the strengths is that it’s there when people need it. I think we are not telling people, ‘You have to be here on this particular day for your session’. We are saying, ‘It’s open. When you need it, it’s right there’” (therapist 19, program B). Furthermore, patients appreciated the absence of time pressure associated with traditional therapy sessions.

I can take time to read the modules. I would never have that luxury to take time and reflect on modules in [a] therapy session...This relaxation of time and availability of resources makes the mental health service delivery much more effective.

Patient 5, program A and program B

### Barriers to iCBT

#### Challenges With the Online Modality

Although therapists recognized the importance of developing a therapeutic alliance with their patients, some noted challenges in establishing rapport and communicating with patients online. Some therapists found it difficult to connect with patients due to the primarily asynchronous nature of communication and the limitations of the messaging platform. For example, 1 therapist noted that “it’s not the same as talking over the phone or in person...you have to wait for them to respond so that can be kind of difficult if they don’t respond...[Also]...it’s not a chat service so it can be hard to maintain a streamlined conversation that you would in person or on the phone because we are supposed to limit our interaction to one to two business days” (therapist 4, program A).

It can be difficult sometimes to build that connection and build that therapeutic relationship when you’re just a voice, or you’re just words on a screen talking to somebody, and you’re not in person getting to build more of that physical or that non-verbal connection as well. So, being online and virtual is definitely very different in terms of how you have to build that relationship as opposed to how you could do it if you were sitting right in front of the person...it just requires a little bit of extra thought into how to develop that relationship when you’re not in person with a patient.

Therapist 8, program A

Therapists were required to refine their skills in connecting with patients through a digital modality.

Despite the reported challenges, some patients said that they were able to develop a strong therapeutic alliance with their therapist online. One patient provided evidence for the ability to develop the therapeutic alliance online, as they stated that “my therapist and I have such a great relationship” (patient 28, program A), while another shared that “I felt understood [and] heard...[the therapist] listened to me...” (patient 26, program A). However, a few patients would have preferred to speak to their therapist over the phone after completing each module. For example, 1 patient stated that they would have preferred to verbally speak to their therapist about their feelings after completing each module, as is their preferred mode of communication: “I would like to express it verbally rather than writing it.” (patient 2, program A)

#### Limited Opportunities for Customization

According to the feedback gathered, patients and providers of the iCBT program felt that it needed more opportunities for customization. They suggested that tailored protocols, feedback, and guidance would enhance the program. While some degree of customization was available through the chat functionality and the ability to add worksheets, tools, or readings, some therapists noted that more needs to be done to make the program more customized to patient needs.

I think the chat functionality, and the ability to add in worksheets or tools or readings at a whim...gives a lot of flexibility, and I think CBT requires more flexibility than is given...we offer reflections, and we ask questions, and sometimes go a little deeper than just what the material presents.

Therapist 20, program B

However, therapists also noted limited options for them to customize the program for their patients, such as the inability to change the patient’s protocol after treatment has started in program B.

I don’t like the fact that when I have assigned a patient a protocol, let’s say, depression, and then we have worked together, and then, maybe, depression is not really the thing I should have assigned this person...but I cannot really change the heading of the treatment.

Therapist 19, program B

Patients also expressed that the lack of tailoring made the program feel “superficial” and not customized to their needs (patient 1, program A). For some patients, the lack of tailoring made them feel “a little boxed into the format” and that the content was “not...necessarily customized for what I [they] needed” (patient 7, program B). Others suggested that given the broad nature of the content, it was not necessarily relatable. They suggested the option for “patient-specific” content based on the patient’s needs (patient 28, program A). One therapist noted that patients have expressed challenges due to the lack of personalization, recognizing that the content has been created in bulk and is not personalized, termed “bulk-therapy” (therapist 10, program B). As 1 therapist stated, many of the messages they sent to their patients were obtained from a bank of prewritten messages, which they would send to their patients after changing the name in the message.

You’re sending messages, but the messages are really short or they’re kind of formulaic...you also need to have the ability to connect with an individual.

Therapist 9, program A

#### Lack of Formal Integration With the Health Care System

The iCBT program was noted to lack formal integration with the health care system by patients and therapists, who expressed a desire for the program to be integrated with other mental health programs and resources. The program currently does not allow therapists to make formal referrals for patients; instead, they can only suggest additional resources, which places the onus on patients to seek other services. Therapists emphasized the need for integration of services, especially given the siloed nature of different services.

We don’t really refer. We make suggestions. We say oh, call this program, or I hear this hospital has an outpatient so-and-so, or you can try so-and-so...I know what referrals are. You fill it in, you phone and you fax it, you confirm, you do handover, and you send case notes. That’s a referral. That’s a solid connection with an appointment usually by the time you’re done for the patient. We don’t do any of that, and we say to the patient it’s all on you.

Therapist, program A

The therapists also suggested that iCBT could be used as a triage process to ensure that patients are matched to the right level of care.

If we’re connected to other mental health agencies, then we can all be able to communicate in a way that’s easy...If this isn’t what would be the best program...we can continue to help you...it’s easy and there’s a feeling that people are really being taken care of.

Therapist 9, program A

They suggested that social workers were in an ideal position to refer patients to other services, but the existing infrastructure does not allow for this.

I would encourage them to reach out to their family doctor [and] provide some resources...and then, I just hope that that’s what’s going to happen...It would be great to have a partnership where the person will have continuity of work, if we could refer them rather than just give them resources for them to self-refer.

Therapist 8, program A

Patients and therapists both felt that patients might still require additional support after completing the iCBT program and desired the ability to stay connected with their therapist on an ad hoc basis after completing the program. Some perceived the loss of contact with the therapist after completing the program to be “abrupt” and expressed the desire for continuity of care with the same provider in the short term or if they signed up for the program again.

## Discussion

The study of patient and therapist perceptions of 2 free publicly funded iCBT programs implemented in Ontario during the COVID-19 pandemic revealed that while several aspects of the program were well-received and facilitated access to care, there were several barriers and missed opportunities. Patients and therapists reported that the following facilitators: (1) the therapist-assisted nature of the program, which enhanced participation, (2) the ease of registration and access, and (3) the online self-paced nature of the program, which increased the feasibility of completing the psychotherapeutic content. However, the study identified three barriers: (1) challenges with the online delivery of the program for developing a therapeutic alliance; (2) the program’s generalized nature, which limited customization to individual needs; and (3) a lack of formal integration between the iCBT program and the health care system.

The findings regarding the facilitators to implementation are largely consistent with what has been reported in the literature. Many patients who were highly engaged with the program felt supported by their therapist and had adequate contact with them, which aligns with previous studies [35]. Furthermore, meta-analyses have found that guided iCBT is more effective than unguided iCBT [36], suggesting that communication with a therapist is one of the active components contributing to the effectiveness of iCBT. Another strength of the program implementation was removing many of the financial, logistical, and emotional barriers for patients. For several clients, the asynchronous communication modality was appreciated as it reduced the barriers associated with stigma for those who were reluctant to engage in traditional therapy formats, many for the first time. These findings are consistent with the literature that posits a key advantage of iCBT is in reducing stigma and, thereby, improving service access [37,38]. Moreover, the absence of a waitlist in both iCBT programs ensured that clients could receive timely access to care, which is crucial given that waitlists are a large barrier to mental health care in Ontario. Across Canada, the average wait time for first-time community mental health services from 2019 to 2020 was nearly 1 month [39]. Last, many patients expressed that the self-paced format of the program allowed them to work through the program at a preferred pace that worked well for their schedule, allowing them to integrate the program into their lives feasibly. These findings are consistent with previous studies of the attitudes of therapists and patients regarding online treatment, for which they positively perceived the ability to perform assignments at the patient’s own time and pace [40,41].

When asked about barriers, some therapists acknowledged challenges in developing the therapeutic alliance with patients; findings that are consistent with other studies in Ontario during the pandemic, where mental health care providers expressed concerns that digital care impeded the therapeutic relationship [42]. However, these findings are contrary to previous studies that found a positive alliance that is similar to face-to-face psychotherapy [43] and with a similar relationship between therapeutic alliance and patient outcomes [44]. Indeed, previous qualitative studies have suggested that the treatment format may act as both a facilitator and barrier to engagement for different individuals [45-49]. While therapists noted that some degree of customization was possible, patients desired more tailored protocols, feedback, and guidance, and some patients expressed that the program felt superficial and not relatable. These findings are aligned with previous qualitative studies on iCBT, where patients desired more flexibility for the therapist to adjust the therapeutic content to their individual difficulties or life situations [50]. While greater customization and therapist involvement may increase effectiveness, there may be a tradeoff of increased costs and, thereby, reduced reach. The most significant finding was that there was a missed opportunity in formally integrating iCBT into the health care system during its implementation, hindering the continuity of care for patients. Furthermore, participants emphasized the need for therapists to provide direct referrals for patients to other services. Understanding for whom specific interventions are most beneficial remains a challenge, particularly for low-intensity interventions such as iCBT [36,51-53]. Last, patients desire continuous support post-iCBT, including necessary follow-up mechanisms and resources for relapse prevention. The frustration experienced by patients who lose access to their therapist has also been reported in qualitative experiences of patients engaging in iCBT [50]. Overall, integrating iCBT with existing services may ensure that adequate treatment options are provided to those for whom iCBT is inappropriate, as well as facilitate the provision of additional support when necessary.

While many iCBT programs have high rates of dropouts or noncompleters [54], the findings provide insight into what works for the minority of patients who are highly engaged with the program. Knowledge and understanding about the patient experience are limited for those with low engagement or who prematurely dropped out of the program. Additionally, despite efforts to reach nonusers, defined as individuals who discontinued treatment after completing the intake assessment but prior to any treatment or were deemed ineligible for the program, no one volunteered to participate in qualitative interviews. Furthermore, the results are not generalizable to all patients and therapists, given the limited variation of sample characteristics and that only those who were interested in sharing their experiences with iCBT chose to participate in the evaluation. Moreover, while the intent of the study was to assess implementation, the interview guide did not include questions to assess users’ perspectives on the perceived impact of the program on their mental health. However, a key strength of this study is that, unlike other real-world implementations of iCBT programs that have been evaluated by those involved in the program development and implementation, the present evaluation was conducted by a neutral third-party evaluation team contracted by the program funder.

The study’s findings have implications for policy, practice, and research. Participants proposed a stepped-care model, wherein the iCBT program could serve as a triage mechanism to ensure that patients are matched with the appropriate level of care. Given the low barriers to access, the program can reach a large segment of the population and provide an entry point into other clinical services that may be more appropriate or act as an introduction to more intensive therapy for patients on waitlists for more specialized services. In clinical practice, iCBT may be used as an introduction to psychotherapy for those who are unsure about traditional CBT, to help prepare clients for more intensive psychotherapy, or when used as a companion to traditional CBT as a booster program following discharge. At the outset of the program implementation, the primary objective was to ensure timely and scalable implementation of iCBT with low barriers to care and broad reach. Understandably, initial decisions regarding the scope and design did not consider how the program could be better integrated into the mental health care system for continuity of care. Policy decisions have since been made based on preliminary findings from a pragmatic evaluation of this program to shift from a low-access model to a prescriptive service offering that requires a referral and is integrated into the Ontario Structured Psychotherapy program, coordinated by 10 partner hospitals across the province [55]. The findings also suggest that iCBT programs could benefit from more personalized approaches to treatment. For example, therapists may benefit from training to be more specific and tailored in their patient feedback rather than using generic responses that may be perceived as disingenuous. To ensure effective support and authenticity in iCBT therapy, further research is needed to determine how therapists can convey these qualities despite the limited communication opportunities in primarily asynchronous care, given the varied patient perceptions of support. In addition, as proposed by Cavanagh et al [56], it may be necessary to reconceptualize the therapeutic alliance in the context of iCBT, given the unique treatment modality, which may guide how therapists are trained to support patients within the context of the program. Furthermore, identifying individuals for whom less therapist interaction is necessary for therapeutic benefit can help allocate more resources for those who may need more frequent interaction and possible modifications to the program structure. However, the discrepancy between the quantitative studies with larger samples and patient experiences reported in qualitative studies suggests that while many patients who do not complete the program report missing face-to-face meetings and synchronous support [46], this may not make a clinical difference. Regardless, future large-scale investigations should examine how iCBT programs can be adjusted to reduce dropout rates for those less satisfied with the treatment format. To enable reaching out to individuals who have dropped out and are no longer actively using the platform, funders should require that vendors provide clients with the option to be contacted outside of the iCBT platform for research purposes by including an additional consent clause during the enrollment process and maintain additional contact details. This is necessary for future research that aims to examine the perspectives of clients who dropped out or disengaged from the program, as they are difficult to reach. Last, the study aims to support an emerging research culture evaluating mobile apps beyond traditional market research done by vendors, wherein therapeutic apps are held to the same standards as other therapeutic interventions, such as drugs, medical devices, or psychotherapy.

In conclusion, our study provides valuable insights into the benefits and limitations of iCBT programs. While the convenience and accessibility of iCBT programs have the potential to be transformative for the treatment of mental health disorders, there is still work to be done to address the concerns of patients and therapists regarding limitations to digital care, customization, and integration with the health care system. Future research should focus on developing more personalized approaches to iCBT treatment, as well as finding ways to better integrate iCBT programs into the existing mental health system.

## Appendix

Multimedia Appendix 1 Interview guide.

## Abbreviations

iCBT: internet-delivered cognitive behavioral therapy
