# Supplementary material for: Patient and Therapist Perceptions of a Publicly Funded Internet-Based Cognitive Behavioral Therapy (iCBT) Program for Ontario Adults During the COVID-19 Pandemic: Qualitative Study
Source: JMIR Form Res. 2024 Feb 19;8:e50113. doi: 10.2196/50113 (PMC10912991; doi:10.2196/50113)
Supplement: Multimedia Appendix 1 [file formative_v8i1e50113_app1.docx]

**User Interview Guide**

**Acceptability (acceptance and satisfaction)**

1. Please tell me about your experience using the iCBT program. (e.g., when did you begin the program, how many modules/playlists did you complete, what did you like most about the program)
2. What made you interested in accessing the iCBT program?
3. How long have you been using the iCBT program?
4. What do you like or dislike about the iCBT program?
5. If you had the option to continue using the iCBT program, would you continue using it? (Tell me more...)

**Appropriateness** **(perceived fit of the program for addressing depression/anxiety-related disorders)**

1. For what reasons did the iCBT program initially seem like a good or appropriate fit for you?
2. After experiencing iCBT, do you feel it was a good match for you in terms of your mental health needs and life situation? (Tell me more about …).
3. How could the iCBT program be improved to better meet your mental health needs? (Tell me more about …).

**Feasibility (enablers and barriers)**

1. Was the iCBT service easy to access? Was it engaging?
2. Was the iCBT program a practical fit in your day-to-day life/workflow? (Tell me more about …).
3. Do you feel realistically able to continue accessing the iCBT program if you wanted to? (Tell me more about …).

**Conclusion**

1. What would need to happen to make the iCBT program meet your mental health needs and particular life situation (e.g., as a working, single parent, as a student, etc.)
2. What other complementary or similar services did you access (if any) alongside iCBT or would like to have accessed, in combination with iCBT (e.g., PCP, counsellor, etc.)?
3. Is there anything else you feel is important to tell us about your experience using the iCBT program?

**Provider Interview Guide**

**Acceptability (acceptance and satisfaction)**

1. Please tell me about your experience delivering the iCBT program.
2. What made you interested in delivering the iCBT program?
3. How long have you been providing care through the iCBT program?
4. What do you like or dislike about the iCBT program?
5. Have there been any concerns about managing or have had problems managing situations of risk (i.e. self-harm) in the iCBT program? Do you plan on continuing to use the program and offering it to patients?
6. How did the iCBT program compare with other forms of therapy you deliver?

**Appropriateness** **(perceived fit of the program for addressing depression/anxiety-related disorders)**

1. What made iCBT a suitable fit / good match for you as a therapist?
2. Are there particular types of clients that benefit the most (or the least) from the program? (Tell me more…) Are there any clients in particular that are better served or not served by iCBT
3. What made iCBT a suitable fit / good match for clients?
4. After delivering assisted therapy through the iCBT program, do you feel it was a good match for you in terms of your clients and practice needs as a health care provider (Tell me more about …).
5. How could the iCBT program be improved to better meet your user and practice needs? (Tell me more about …).

**Feasibility (enablers and barriers)**

1. Was it easy to use the iCBT program and deliver assisted therapy through it to patients?
2. What kind of administrative/technological support did you have available to you, if any, in delivering assisted therapy through the iCBT program?
3. Did you receive any feedback from patients in terms of their perceptions about how easy or difficult the iCBT program was to use/access?
4. What kinds of challenges did patients face?
5. What is your clinical perspective on the self-referral assessment and process of the program?
6. What kind of professional development opportunities or collegial support did you have available to you, if any, in delivering assisted therapy through the iCBT program?
7. Was the iCBT program a practical fit in your day-to-day practice workflow? (Tell me more about …).

**Conclusion**

1. What would need to happen to make the iCBT program more tailored to your clients’ needs?
2. What would need to happen to make the iCBT program more tailored for your needs as a health care provider in your practice setting?
3. Are there any services that you think would need to be added or be complementary to include with the iCBT program?
4. Is there anything else you feel is important to tell us about your experience using the iCBT program?
